# Supplementary material for: Phenotypic proteomic profiling identifies a landscape of targets for circadian clock–modulating compounds
Source: Life Sci Alliance. 2019 Dec 2;2(6):e201900603. doi: 10.26508/lsa.201900603 (PMC6892409; doi:10.26508/lsa.201900603)
Supplement: Supplementary file 1 [file LSA-2019-00603_TableS1.docx]

**Table S1.** Alterations in U2OS proteome induced by circadian period lengthening drugs

**Table S1. A**. Differentially expressed proteins (*p_Adj_* < 0.05) identified in U2OS cells after Longdaysin treatment

| SL NO | Accession | Gene Name | Protein Name | Average Fold-change | *p_Adj_* value |
| --- | --- | --- | --- | --- | --- |
| 1 | P62805 | *Hist1h4a* | Histone H4 | 1.36 | 0.012 |
| 2 | P07305 | *H1f0* | Histone H1.0 | 1.31 | 0.004 |
| 3 | Q71DI3 | *Hist2h3a* | Histone H3.2 | 1.28 | 0.011 |
| 4 | Q9H1E3 | *Nucks1* | Nuclear ubiquitous casein and cyclin-dependent kinase substrate 1 | 1.22 | 0.012 |
| 5 | P61916 | *Npc2* | Epididymal secretory protein E1 | 1.20 | 0.0001 |
| 6 | P07858 | *Ctsb* | Cathepsin B | 1.19 | 0.009 |
| 7 | Q16611 | *Bak1* | Bcl-2 homologous antagonist/killer | 1.18 | 0.034 |
| 8 | P08236 | *Gusb* | Beta-glucuronidase | 1.17 | 0.023 |
| 9 | O43169 | *Cyb5b* | Cytochrome b5 type B | 1.15 | 0.042 |
| 10 | Q15554 | *Terf2* | Telomeric repeat-binding factor 2 | 1.15 | 0.019 |
| 11 | Q13427 | *Ppig* | Peptidyl-prolyl cis-trans isomerase G | 0.89 | 0.040 |
| 12 | P61513 | *Rpl37a* | 60S ribosomal protein L37a | 0.88 | 0.024 |
| 13 | P56381 | *Atp5f1e* | ATP synthase subunit epsilon, mitochondrial | 0.88 | 0.050 |
| 14 | Q92925 | *Smarcd2* | SWI/SNF-related matrix-associated actin-dependent regulator of chromatin subfamily D member 2 | 0.88 | 0.035 |
| 15 | P61927 | *Rpl37* | 60S ribosomal protein L37 | 0.87 | 0.028 |
| 16 | Q7Z589 | *Emsy* | Protein EMSY | 0.86 | 0.011 |
| 17 | Q9Y305 | *Acot9* | Acyl-coenzyme A thioesterase 9, mitochondrial | 0.86 | 0.044 |
| 18 | P46779 | *Rpl28* | 60S ribosomal protein L28 | 0.86 | 0.008 |
| 19 | Q07020 | *Rpl18* | 60S ribosomal protein L18 | 0.85 | 0.001 |
| 20 | P50613 | *Cdk7* | Cyclin-dependent kinase 7 | 0.85 | 0.009 |
| 21 | P62424 | *Rpl7a* | 60S ribosomal protein L7a | 0.85 | 0.003 |
| 22 | P02452 | *Col1a1* | Collagen alpha-1(I) chain | 0.83 | 0.0001 |
| 23 | P19784 | *Csnk2a2* | Casein kinase II subunit alpha' | 0.81 | 0.039 |
| 24 | P50914 | *Rpl14* | 60S ribosomal protein L14 | 0.80 | 0.008 |
| 25 | O60524 | *Nemf* | Nuclear export mediator factor NEMF | 0.80 | 0.029 |
| 26 | Q16539 | *Mapk14* | Mitogen-activated protein kinase 14 | 0.78 | 0.0002 |
| 27 | Q8WW59 | *Spryd4* | SPRY domain-containing protein 4 | 0.78 | 0.035 |
| 28 | P35527 | *Krt9* | Keratin, type I cytoskeletal 9 | 0.78 | 0.049 |
| 29 | P48730 | *Csnk1d* | Casein kinase I isoform delta | 0.75 | 0.00003 |
| 30 | P28482 | *Mapk1* | Mitogen-activated protein kinase 1 | 0.75 | 0.00035 |
| 31 | Q9NZ01 | *Tecr* | Very-long-chain enoyl-CoA reductase | 0.46 | 0.00004 |

**Table S1. B**. Differentially expressed proteins (*p_Adj_* < 0.05) identified in U2OS cells after Purvalanol A treatment

| SL NO | Accession | Gene Name | Protein Name | Average Fold-change | *p_Adj_* value |
| --- | --- | --- | --- | --- | --- |
| 1 | P38936 | *Cdkn1a* | Cyclin-dependent kinase inhibitor 1 | 1.52 | 0.0004 |
| 2 | P62805 | *Hist1h4a* | Histone H4 | 1.50 | 0.006 |
| 3 | P46527 | *Cdkn1b* | Cyclin-dependent kinase inhibitor 1B | 1.37 | 0.004 |
| 4 | P61916 | *Npc2* | Epididymal secretory protein E1 | 1.33 | 0.0003 |
| 5 | P29034 | *S100a2* | Protein S100-A2 | 1.30 | 0.00001 |
| 6 | O95810 | *Cavin2* | Serum deprivation-response protein | 1.29 | 0.0002 |
| 7 | Q71DI3 | *Hist2h3a;* | Histone H3.2 | 1.29 | 0.020 |
| 8 | P07858 | *Ctsb* | Cathepsin B | 1.29 | 0.00001 |
| 9 | O75528 | *Tada3* | Transcriptional adapter 3 | 1.28 | 0.021 |
| 10 | Q6NZI2 | *Cavin1* | Polymerase I and transcript release factor | 1.22 | 0.002 |
| 11 | P13807 | *Gys1* | Glycogen [starch] synthase, muscle | 1.22 | 0.027 |
| 12 | P07602 | *Psap* | Prosaposin | 1.20 | 0.001 |
| 13 | Q8WUM9 | *Slc20a1* | Sodium-dependent phosphate transporter 1 | 1.20 | 0.027 |
| 14 | P07305 | *H1f0* | Histone H1.0 | 1.20 | 0.005 |
| 15 | Q13740 | *Alcam* | CD166 antigen | 1.20 | 0.002 |
| 16 | P17301 | *Itga2* | Integrin alpha-2 | 1.20 | 0.003 |
| 17 | Q13153 | *Pak1* | Serine/threonine-protein kinase PAK 1 | 1.19 | 0.038 |
| 18 | P17050 | *Naga* | Alpha-N-acetylgalactosaminidase | 1.19 | 0.025 |
| 19 | Q52LW3 | *Arhgap29* | Rho GTPase-activating protein 29 | 1.19 | 0.025 |
| 20 | P25445 | *Fas* | Tumor necrosis factor receptor superfamily member 6 | 1.18 | 0.032 |
| 21 | Q9NRX5 | *Serinc1* | Serine incorporator 1 | 1.18 | 0.011 |
| 22 | Q08722 | *Cd47* | Leukocyte surface antigen CD47 | 1.17 | 0.018 |
| 23 | P26006 | *Itga3* | Integrin alpha-3 | 1.17 | 0.013 |
| 24 | Q03135 | *Cav1* | Caveolin-1 | 1.17 | 0.002 |
| 25 | P53794 | *Slc5a3* | Sodium/myo-inositol cotransporter | 1.16 | 0.034 |
| 26 | Q96CM8 | *Acsf2* | Acyl-CoA synthetase family member 2, mitochondrial | 1.16 | 0.001 |
| 27 | P17096 | *Hmga1* | High mobility group protein HMG-I/HMG-Y | 1.16 | 0.002 |
| 28 | Q9H1B7 | *Irf2bpl* | Interferon regulatory factor 2-binding protein-like | 1.15 | 0.039 |
| 29 | P05204 | *Hmgn2* | Non-histone chromosomal protein HMG-17 | 1.15 | 0.012 |
| 30 | O14531 | *Dpysl4* | Dihydropyrimidinase-related protein 4 | 1.15 | 0.041 |
| 31 | Q8IZQ5 | *Selenoh* | Selenoprotein H | 1.14 | 0.017 |
| 32 | Q8N5B7 | *Cers5* | Ceramide synthase 5 | 1.14 | 0.040 |
| 33 | Q00534 | *Cdk6* | Cyclin-dependent kinase 6 | 0.86 | 0.024 |
| 34 | P61024 | *Cks1b* | Cyclin-dependent kinases regulatory subunit 1 | 0.85 | 0.049 |
| 35 | P36873 | *Ppp1cc* | Serine/threonine-protein phosphatase PP1-gamma catalytic subunit | 0.85 | 0.022 |
| 36 | O60934 | *Nbn* | Nibrin | 0.83 | 0.029 |
| 37 | P33552 | *Cks2* | Cyclin-dependent kinases regulatory subunit 2 | 0.83 | 0.031 |
| 38 | Q96T88 | *Uhrf1* | E3 ubiquitin-protein ligase UHRF1 | 0.82 | 0.014 |
| 39 | P02452 | *Col1a1* | Collagen alpha-1(I) chain | 0.80 | 0.014 |
| 40 | O94925 | *Gls* | Glutaminase kidney isoform, mitochondrial | 0.80 | 0.003 |
| 41 | Q01581 | *Hmgcs1* | Hydroxymethylglutaryl-CoA synthase, cytoplasmic | 0.77 | 0.0001 |
| 42 | P50914 | *Rpl14* | 60S ribosomal protein L14 | 0.75 | 0.00002 |
| 43 | P50613 | *Cdk7* | Cyclin-dependent kinase 7 | 0.74 | 0.012 |

| SL NO | Accession | Gene Name | Protein Name | Average Fold-change | *p_Adj_* value |
| --- | --- | --- | --- | --- | --- |
| 1 | Q71DI3 | *Hist2h3a* | Histone H3.2 | 1.34 | 0.011 |
| 2 | P01023 | *A2m* | Alpha-2-macroglobulin | 1.32 | 0.020 |
| 3 | P38936 | *Cdkn1a* | Cyclin-dependent kinase inhibitor 1 | 1.32 | 0.0002 |
| 4 | P62805 | *Hist1h4a* | Histone H4 | 1.32 | 0.021 |
| 5 | P02452 | *Col1a1* | Collagen alpha-1(I) chain | 1.23 | 0.012 |
| 6 | Q92542 | *Ncstn* | Nicastrin | 1.22 | 0.010 |
| 7 | Q9Y5Y2 | *Nubp2* | Cytosolic Fe-S cluster assembly factor NUBP2 | 1.22 | 0.029 |
| 8 | P16104 | *H2afx* | Histone H2AX | 1.21 | 0.019 |
| 9 | P13807 | *Gys1* | Glycogen [starch] synthase, muscle | 1.20 | 0.003 |
| 10 | Q13153 | *Pak1* | Serine/threonine-protein kinase PAK 1 | 1.19 | 0.031 |
| 11 | Q9P2T1 | *Gmpr2* | GMP reductase 2 | 1.18 | 0.038 |
| 12 | Q53H96 | *Pycr3* | Pyrroline-5-carboxylate reductase 3 | 1.15 | 0.033 |
| 13 | O14519 | *Cdk2ap1* | Cyclin-dependent kinase 2-associated protein 1 | 1.15 | 0.007 |
| 14 | P32322 | *Pycr1* | Pyrroline-5-carboxylate reductase 1, mitochondrial | 1.15 | 0.024 |
| 15 | Q8NHP8 | *Plbd2* | Putative phospholipase B-like 2 | 1.15 | 0.033 |
| 16 | P15529 | *Cd46* | Membrane cofactor protein | 1.14 | 0.041 |
| 17 | Q8WUM9 | *Slc20a1* | Sodium-dependent phosphate transporter 1 | 1.14 | 0.045 |
| 18 | A8MWD9 | *Snrpgp15* | Small nuclear ribonucleoprotein G-like protein | 1.14 | 0.031 |
| 19 | P52926 | *Hmga2* | High mobility group protein HMGI-C | 1.13 | 0.019 |
| 20 | Q9NX74 | *Dus2* | tRNA-dihydrouridine(20) synthase [NAD(P)+]-like | 1.13 | 0.034 |
| 21 | Q92576 | *Phf3* | PHD finger protein 3 | 1.13 | 0.022 |
| 22 | Q14527 | *Hltf* | Helicase-like transcription factor | 0.89 | 0.047 |
| 23 | P26373 | *Rpl13* | 60S ribosomal protein L13 | 0.88 | 0.028 |
| 24 | P30453 | *Hla-a* | HLA class I histocompatibility antigen, A-34 alpha chain | 0.87 | 0.025 |
| 25 | O76021 | *Rsl1d1* | Ribosomal L1 domain-containing protein 1 | 0.87 | 0.003 |
| 26 | P21127 | *Cdk11b* | Cyclin-dependent kinase 11B | 0.86 | 0.008 |
| 27 | P49207 | *Rpl34* | 60S ribosomal protein L34 | 0.86 | 0.001 |
| 28 | P83731 | *Rpl24* | 60S ribosomal protein L24 | 0.85 | 0.004 |
| 29 | Q9H1E3 | *Nucks1* | Nuclear ubiquitous casein and cyclin-dependent kinase substrate 1 | 0.85 | 0.011 |
| 30 | P62424 | *Rpl7a* | 60S ribosomal protein L7a | 0.84 | 0.015 |
| 31 | O14979 | *Hnrnpdl* | Heterogeneous nuclear ribonucleoprotein D-like | 0.84 | 0.0003 |
| 32 | O60524 | *Nemf* | Nuclear export mediator factor NEMF | 0.84 | 0.024 |
| 33 | Q07020 | *Rpl18* | 60S ribosomal protein L18 | 0.83 | 0.001 |
| 34 | P11388 | *Top2a* | DNA topoisomerase 2-alpha | 0.83 | 0.029 |
| 35 | P61353 | *Rpl27* | 60S ribosomal protein L27 | 0.83 | 0.010 |
| 36 | P61024 | *Cks1b* | Cyclin-dependent kinases regulatory subunit 1 | 0.82 | 0.027 |
| 37 | P50613 | *Cdk7* | Cyclin-dependent kinase 7 | 0.81 | 0.0004 |
| 38 | P50750 | *Cdk9* | Cyclin-dependent kinase 9 | 0.81 | 0.014 |
| 39 | P24941 | *Cdk2* | Cyclin-dependent kinase 2 | 0.81 | 0.003 |
| 40 | P06493 | *Cdk1* | Cyclin-dependent kinase 1 | 0.80 | 0.029 |
| 41 | Q00534 | *Cdk6* | Cyclin-dependent kinase 6 | 0.80 | 0.00012 |
| 42 | P54652 | *Hspa2* | Heat shock-related 70 kDa protein 2 | 0.79 | 0.00004 |
| 43 | O94992 | *Hexim1* | Protein HEXIM1 | 0.79 | 0.011 |
| 44 | P50914 | *Rpl14* | 60S ribosomal protein L14 | 0.75 | 0.0001 |

**Table S1. C**. Differentially expressed proteins (*p_Adj_* < 0.05) identified in U2OS cells after Roscovitine treatment

**Table S1. D**. Differentially expressed proteins (*p_Adj_* < 0.05) identified in U2OS cells after SP600125 treatment

| SL NO | Accession | Gene Name | Protein Name | Average Fold-change | *p_Adj_* value |
| --- | --- | --- | --- | --- | --- |
| 1 | P02768 | *Alb* | Serum albumin | 4.92 | 0.006 |
| 2 | P62805 | *Hist1h4a* | Histone H4 | 2.04 | 0.002 |
| 3 | Q9Y6V0 | *Pclo* | Protein piccolo | 1.68 | 0.015 |
| 4 | Q9Y3T9 | *Noc2l* | Nucleolar complex protein 2 homolog | 1.62 | 0.012 |
| 5 | Q71DI3 | *Hist2h3a* | Histone H3.2 | 1.59 | 0.003 |
| 6 | P34059 | *Galns* | N-acetylgalactosamine-6-sulfatase | 1.33 | 0.027 |
| 7 | P07305 | *H1f0* | Histone H1.0 | 1.33 | 0.003 |
| 8 | P49711 | *Ctcf* | Transcriptional repressor CTCF | 1.25 | 0.012 |
| 9 | P05204 | *Hmgn2* | Non-histone chromosomal protein HMG-17 | 1.22 | 0.003 |
| 10 | O95810 | *Cavin2* | Serum deprivation-response protein | 1.22 | 0.0004 |
| 11 | Q92576 | *Phf3* | PHD finger protein 3 | 1.21 | 0.002 |
| 12 | Q9NWH9 | *Sltm* | SAFB-like transcription modulator | 1.21 | 0.006 |
| 13 | O75528 | *Tada3* | Transcriptional adapter 3 | 1.20 | 0.016 |
| 14 | Q8IYM9 | *Trim22* | E3 ubiquitin-protein ligase TRIM22 | 1.19 | 0.016 |
| 15 | P04179 | *Sod2* | Superoxide dismutase [Mn], mitochondrial | 1.18 | 0.001 |
| 16 | Q96RQ1 | *Ergic2* | Endoplasmic reticulum-Golgi intermediate compartment protein 2 | 1.18 | 0.027 |
| 17 | P52926 | *Hmga2* | High mobility group protein HMGI-C | 1.16 | 0.044 |
| 18 | P63173 | *Rpl38* | 60S ribosomal protein L38 | 0.89 | 0.051 |
| 19 | Q9NVM6 | *Dnajc17* | DnaJ homolog subfamily C member 17 | 0.88 | 0.017 |
| 20 | Q2NL82 | *Tsr1* | Pre-rRNA-processing protein TSR1 homolog | 0.87 | 0.032 |
| 21 | Q9H3P2 | *Nelfa* | Negative elongation factor A | 0.86 | 0.038 |
| 22 | O76021 | *Rsl1d1* | Ribosomal L1 domain-containing protein 1 | 0.86 | 0.005 |
| 23 | P62917 | *Rpl8* | 60S ribosomal protein L8 | 0.85 | 0.0004 |
| 24 | Q92597 | *Ndrg1* | Protein NDRG1 | 0.85 | 0.032 |
| 25 | P62424 | *Rpl7a* | 60S ribosomal protein L7a | 0.85 | 0.008 |
| 26 | Q99538 | *Lgmn* | Legumain | 0.84 | 0.004 |
| 27 | O15460 | *P4ha2* | Prolyl 4-hydroxylase subunit alpha-2 | 0.84 | 0.00001 |
| 28 | Q9GZP8 | *Imup* | Immortalization up-regulated protein | 0.84 | 0.034 |
| 29 | P02511 | *Cryab* | Alpha-crystallin B chain | 0.84 | 0.002 |
| 30 | P42766 | *Rpl35* | 60S ribosomal protein L35 | 0.84 | 0.009 |
| 31 | Q00534 | *Cdk6* | Cyclin-dependent kinase 6 | 0.83 | 0.016 |
| 32 | P61254 | *Rpl26* | 60S ribosomal protein L26 | 0.83 | 0.00001 |
| 33 | P47914 | *Rpl29* | 60S ribosomal protein L29 | 0.83 | 0.013 |
| 34 | Q8TBX8 | *Pip4k2c* | Phosphatidylinositol 5-phosphate 4-kinase type-2 gamma | 0.83 | 0.022 |
| 35 | P26373 | *Rpl13* | 60S ribosomal protein L13 | 0.82 | 0.00003 |
| 36 | Q07020 | *Rpl18* | 60S ribosomal protein L18 | 0.82 | 0.014 |
| 37 | P32004 | *L1cam* | Neural cell adhesion molecule L1 | 0.82 | 0.00005 |
| 38 | P04732 | *Mt1e* | Metallothionein-1E | 0.81 | 0.039 |
| 39 | P62753 | *Rps6* | 40S ribosomal protein S6 | 0.81 | 0.0002 |
| 40 | P61353 | *Rpl27* | 60S ribosomal protein L27 | 0.81 | 0.0001 |
| 41 | O60524 | *Nemf* | Nuclear export mediator factor NEMF | 0.81 | 0.0004 |
| 42 | O94925 | *Gls* | Glutaminase kidney isoform, mitochondrial | 0.81 | 0.000001 |
| 43 | P11387 | *Top1* | DNA topoisomerase 1 | 0.81 | 0.015 |
| 44 | P36578 | *Rpl4* | 60S ribosomal protein L4 | 0.80 | 0.0002 |
| 45 | O60271 | *Spag9* | C-Jun-amino-terminal kinase-interacting protein 4 | 0.80 | 0.005 |
| 46 | P62847 | *Rps24* | 40S ribosomal protein S24 | 0.79 | 0.013 |
| 47 | P17535 | *Jund* | Transcription factor jun-D | 0.78 | 0.0000001 |
| 48 | P05412 | *Jun* | Transcription factor AP-1 | 0.78 | 0.009 |
| 49 | Q02878 | *Rpl6* | 60S ribosomal protein L6 | 0.78 | 0.0003 |
| 50 | Q8WW59 | *Spryd4* | SPRY domain-containing protein 4 | 0.78 | 0.039 |
| 51 | P83731 | *Rpl24* | 60S ribosomal protein L24 | 0.76 | 0.0001 |
| 52 | P50914 | *Rpl14* | 60S ribosomal protein L14 | 0.74 | 0.004 |
| 53 | P29034 | *S100a2* | Protein S100-A2 | 0.74 | 0.0001 |
| 54 | P49207 | *Rpl34* | 60S ribosomal protein L34 | 0.72 | 0.001 |
